# Supplementary material for: Hot spot prediction in protein-protein interactions by an ensemble system
Source: BMC Syst Biol. 2018 Dec 31;12(Suppl 9):132. doi: 10.1186/s12918-018-0665-8 (PMC6311905; doi:10.1186/s12918-018-0665-8)
Supplement: Supplementary file 2 — All relevant MATLAB code. A simple Matlab implement of our predictor. (ZIP 19,742 kb) [file 12918_2018_665_MOESM2_ESM.zip › Additional file 2/Read ASA/ASA website/BID/1JATA.htm]

 NetSurfP - Protein Surface Accessibility and Secondary Structure Predictions

  

|  |  |  |
| --- | --- | --- |
|  |  | NetSurfP - Protein Surface Accessibility and Secondary Structure Predictions  Technical University of Denmark |

  


---

```
# For publication of results, please cite:
# A generic method for assignment of reliability scores applied to solvent accessibility predictions.
# Bent Petersen, Thomas Nordahl Petersen, Pernille Andersen, Morten Nielsen and Claus Lundegaard
# BMC Structural Biology 2009, 9:51 doi:10.1186/1472-6807-9-51
#
# Column 1: Class assignment - B for buried or E for Exposed - Threshold: 25% exposure, but not based on RSA
# Column 2: Amino acid
# Column 3: Sequence name
# Column 4: Amino acid number
# Column 5: Relative Surface Accessibility - RSA
# Column 6: Absolute Surface Accessibility
# Column 7: Z-fit score for RSA prediction
# Column 8: Probability for Alpha-Helix
# Column 9: Probability for Beta-strand
# Column 10: Probability for Coil
E G  1JATA                  1    0.885  69.626  -1.847   0.000   0.001   0.999
E S  1JATA                  2    0.561  65.784  -2.257   0.115   0.016   0.868
E A  1JATA                  3    0.515  56.764  -2.011   0.600   0.003   0.397
E A  1JATA                  4    0.536  59.078  -0.436   0.782   0.003   0.216
E S  1JATA                  5    0.469  54.967   0.143   0.923   0.002   0.076
B L  1JATA                  6    0.120  21.972  -0.502   0.923   0.002   0.076
B P  1JATA                  7    0.274  38.923  -0.006   0.970   0.001   0.030
E K  1JATA                  8    0.474  97.522   0.935   0.970   0.001   0.030
B R  1JATA                  9    0.239  54.685   1.357   0.970   0.001   0.030
B I  1JATA                 10    0.044   8.048   0.591   0.970   0.001   0.030
E I  1JATA                 11    0.271  50.190   1.364   0.970   0.001   0.030
E K  1JATA                 12    0.481  98.983   1.623   0.970   0.001   0.030
B E  1JATA                 13    0.084  14.692   0.324   0.970   0.001   0.030
B T  1JATA                 14    0.096  13.329   0.835   0.988   0.000   0.012
E E  1JATA                 15    0.479  83.734   1.544   0.970   0.001   0.030
E K  1JATA                 16    0.463  95.198   2.113   0.970   0.001   0.030
B L  1JATA                 17    0.047   8.624   0.459   0.923   0.002   0.076
E V  1JATA                 18    0.429  65.891   0.070   0.782   0.003   0.216
E S  1JATA                 19    0.712  83.400   0.218   0.502   0.002   0.495
E D  1JATA                 20    0.567  81.777  -1.414   0.053   0.005   0.942
E P  1JATA                 21    0.483  68.524  -0.888   0.003   0.003   0.994
B V  1JATA                 22    0.285  43.835  -1.555   0.005   0.015   0.979
E P  1JATA                 23    0.490  69.517  -1.256   0.005   0.015   0.979
B G  1JATA                 24    0.201  15.834  -0.728   0.005   0.262   0.733
B I  1JATA                 25    0.023   4.274   0.742   0.002   0.816   0.182
B T  1JATA                 26    0.164  22.761   0.386   0.001   0.959   0.040
B A  1JATA                 27    0.026   2.810  -0.276   0.001   0.959   0.040
B E  1JATA                 28    0.179  31.254   0.715   0.001   0.900   0.099
B P  1JATA                 29    0.098  13.864  -0.181   0.003   0.718   0.279
E H  1JATA                 30    0.318  57.790   0.156   0.005   0.336   0.660
E D  1JATA                 31    0.586  84.399  -1.578   0.005   0.045   0.951
E D  1JATA                 32    0.692  99.674  -1.466   0.018   0.019   0.964
E N  1JATA                 33    0.262  38.386   0.125   0.018   0.088   0.893
B L  1JATA                 34    0.096  17.486   0.483   0.021   0.451   0.528
B R  1JATA                 35    0.155  35.541   0.200   0.004   0.616   0.381
B Y  1JATA                 36    0.181  38.787   1.095   0.001   0.900   0.099
B F  1JATA                 37    0.022   4.355   1.285   0.001   0.959   0.040
B Q  1JATA                 38    0.116  20.807   0.947   0.000   0.983   0.017
B V  1JATA                 39    0.013   1.921   1.221   0.000   0.983   0.017
B T  1JATA                 40    0.085  11.776   0.679   0.000   0.983   0.017
B I  1JATA                 41    0.023   4.163   1.028   0.000   0.983   0.017
B E  1JATA                 42    0.164  28.581   0.710   0.001   0.959   0.040
B G  1JATA                 43    0.077   6.036  -0.725   0.003   0.718   0.279
E P  1JATA                 44    0.371  52.645  -0.159   0.005   0.336   0.660
E E  1JATA                 45    0.676 118.167  -1.473   0.004   0.085   0.910
E Q  1JATA                 46    0.683 121.966  -1.611   0.005   0.015   0.979
B S  1JATA                 47    0.177  20.768  -1.258   0.005   0.045   0.951
B P  1JATA                 48    0.223  31.644  -0.237   0.004   0.197   0.799
B Y  1JATA                 49    0.090  19.254  -0.404   0.005   0.262   0.733
E E  1JATA                 50    0.462  80.729  -1.088   0.004   0.197   0.799
E D  1JATA                 51    0.543  78.261  -1.661   0.004   0.138   0.858
B G  1JATA                 52    0.139  10.939  -1.130   0.004   0.514   0.481
B I  1JATA                 53    0.237  43.808   0.305   0.001   0.900   0.099
B F  1JATA                 54    0.053  10.617   0.699   0.001   0.959   0.040
B E  1JATA                 55    0.219  38.224   1.240   0.001   0.959   0.040
B L  1JATA                 56    0.022   4.028   0.987   0.001   0.959   0.040
B E  1JATA                 57    0.191  33.438   1.044   0.001   0.959   0.040
B L  1JATA                 58    0.036   6.518  -0.009   0.001   0.959   0.040
E Y  1JATA                 59    0.226  48.189   0.525   0.001   0.900   0.099
B L  1JATA                 60    0.029   5.365   0.313   0.003   0.718   0.279
B P  1JATA                 61    0.195  27.713   0.240   0.004   0.138   0.858
E D  1JATA                 62    0.456  65.782  -1.081   0.018   0.019   0.964
E D  1JATA                 63    0.608  87.570  -1.328   0.018   0.019   0.964
B Y  1JATA                 64    0.129  27.460   0.325   0.018   0.019   0.964
E P  1JATA                 65    0.303  43.038  -1.409   0.018   0.047   0.935
B M  1JATA                 66    0.296  59.210  -1.417   0.004   0.085   0.910
E E  1JATA                 67    0.334  58.332  -0.839   0.004   0.138   0.858
B A  1JATA                 68    0.144  15.858   0.219   0.004   0.197   0.799
B P  1JATA                 69    0.057   8.088  -0.382   0.004   0.514   0.481
B K  1JATA                 70    0.192  39.515   0.205   0.002   0.816   0.182
B V  1JATA                 71    0.019   2.951   0.837   0.001   0.900   0.099
B R  1JATA                 72    0.129  29.541   0.221   0.001   0.959   0.040
B F  1JATA                 73    0.025   5.078  -0.585   0.001   0.959   0.040
B L  1JATA                 74    0.090  16.424  -0.399   0.002   0.816   0.182
B T  1JATA                 75    0.046   6.352  -0.901   0.004   0.420   0.576
B K  1JATA                 76    0.136  28.016  -0.416   0.021   0.279   0.699
B I  1JATA                 77    0.052   9.657  -1.197   0.005   0.336   0.660
B Y  1JATA                 78    0.071  15.130  -2.827   0.004   0.420   0.576
B H  1JATA                 79    0.045   8.131  -0.735   0.004   0.138   0.858
B P  1JATA                 80    0.073  10.345  -3.616   0.018   0.047   0.935
B N  1JATA                 81    0.116  17.012  -3.128   0.018   0.088   0.893
B I  1JATA                 82    0.121  22.348  -2.142   0.021   0.279   0.699
B D  1JATA                 83    0.248  35.780  -1.011   0.020   0.205   0.775
E R  1JATA                 84    0.508 116.309  -1.997   0.018   0.047   0.935
E L  1JATA                 85    0.507  92.777  -2.077   0.005   0.015   0.979
B G  1JATA                 86    0.169  13.316  -2.581   0.018   0.088   0.893
E R  1JATA                 87    0.301  68.860  -1.111   0.022   0.359   0.619
B I  1JATA                 88    0.097  17.945  -0.579   0.022   0.552   0.426
B C  1JATA                 89    0.064   9.042  -0.947   0.023   0.655   0.322
B L  1JATA                 90    0.187  34.276  -0.421   0.021   0.451   0.528
E D  1JATA                 91    0.338  48.634  -1.733   0.064   0.216   0.721
E V  1JATA                 92    0.345  53.042  -0.392   0.052   0.084   0.864
B L  1JATA                 93    0.171  31.328  -0.873   0.053   0.043   0.903
E K  1JATA                 94    0.430  88.554  -0.396   0.058   0.017   0.925
E T  1JATA                 95    0.738 102.291  -0.925   0.058   0.017   0.925
E N  1JATA                 96    0.544  79.598  -0.484   0.018   0.019   0.964
B W  1JATA                 97    0.064  15.320  -1.151   0.018   0.019   0.964
E S  1JATA                 98    0.367  43.024   0.354   0.058   0.017   0.925
E P  1JATA                 99    0.344  48.785  -0.576   0.257   0.016   0.727
E A  1JATA                100    0.433  47.684  -1.341   0.339   0.016   0.645
B L  1JATA                101    0.203  37.243   0.537   0.268   0.043   0.689
E Q  1JATA                102    0.275  49.097   0.361   0.354   0.048   0.598
B I  1JATA                103    0.033   6.105  -0.395   0.879   0.010   0.111
E R  1JATA                104    0.282  64.509   0.473   0.970   0.001   0.030
E T  1JATA                105    0.284  39.446   0.765   0.970   0.001   0.030
B V  1JATA                106    0.023   3.566   1.154   0.970   0.001   0.030
B L  1JATA                107    0.038   6.903   0.015   0.970   0.001   0.030
E L  1JATA                108    0.252  46.141   0.700   0.970   0.001   0.030
B S  1JATA                109    0.079   9.200  -0.269   0.970   0.001   0.030
B I  1JATA                110    0.016   2.941   1.451   0.988   0.000   0.012
B Q  1JATA                111    0.158  28.165   0.426   0.988   0.000   0.012
E A  1JATA                112    0.265  29.170  -0.411   0.970   0.001   0.030
B L  1JATA                113    0.039   7.104  -0.271   0.923   0.002   0.076
B L  1JATA                114    0.028   5.054  -0.297   0.782   0.003   0.216
E A  1JATA                115    0.342  37.699  -0.306   0.600   0.003   0.397
E S  1JATA                116    0.388  45.474  -0.271   0.246   0.004   0.750
B P  1JATA                117    0.227  32.225  -0.701   0.109   0.005   0.886
E N  1JATA                118    0.308  45.106  -0.501   0.016   0.005   0.979
B P  1JATA                119    0.270  38.341  -1.075   0.053   0.005   0.942
E N  1JATA                120    0.711 104.046  -0.937   0.016   0.005   0.979
E D  1JATA                121    0.283  40.809  -0.918   0.016   0.005   0.979
B P  1JATA                122    0.130  18.433  -1.972   0.176   0.004   0.820
B L  1JATA                123    0.142  26.019  -1.173   0.181   0.016   0.803
B A  1JATA                124    0.250  27.594  -0.163   0.339   0.016   0.645
E N  1JATA                125    0.332  48.619  -0.301   0.923   0.002   0.076
E D  1JATA                126    0.384  55.334  -0.337   0.923   0.002   0.076
B V  1JATA                127    0.033   5.118  -0.584   0.970   0.001   0.030
B A  1JATA                128    0.084   9.246  -0.459   0.970   0.001   0.030
E E  1JATA                129    0.465  81.253   1.191   0.970   0.001   0.030
B D  1JATA                130    0.147  21.125   0.592   0.923   0.002   0.076
B W  1JATA                131    0.120  28.860   0.832   0.858   0.002   0.139
E I  1JATA                132    0.462  85.470   1.318   0.782   0.003   0.216
E K  1JATA                133    0.616 126.691   0.744   0.600   0.003   0.397
E N  1JATA                134    0.483  70.653   0.275   0.053   0.005   0.942
E E  1JATA                135    0.357  62.333   0.894   0.923   0.002   0.076
E Q  1JATA                136    0.703 125.538   1.344   0.970   0.001   0.030
E G  1JATA                137    0.415  32.645   1.725   0.970   0.001   0.030
B A  1JATA                138    0.036   3.923  -0.333   0.970   0.001   0.030
E K  1JATA                139    0.254  52.248   1.313   0.988   0.000   0.012
E A  1JATA                140    0.529  58.296   1.209   0.970   0.001   0.030
B K  1JATA                141    0.244  50.273   1.851   0.988   0.000   0.012
B A  1JATA                142    0.011   1.234   1.858   0.988   0.000   0.012
E R  1JATA                143    0.259  59.265   1.807   0.988   0.000   0.012
E E  1JATA                144    0.462  80.746   1.671   0.988   0.000   0.012
B W  1JATA                145    0.101  24.218   1.233   0.988   0.000   0.012
B T  1JATA                146    0.022   3.010   0.900   0.988   0.000   0.012
E K  1JATA                147    0.437  89.891   1.154   0.970   0.001   0.030
E L  1JATA                148    0.492  90.012   1.671   0.923   0.002   0.076
B Y  1JATA                149    0.161  34.491   0.099   0.858   0.002   0.139
B A  1JATA                150    0.134  14.767  -0.260   0.406   0.004   0.590
E K  1JATA                151    0.577 118.648   0.524   0.246   0.004   0.750
E K  1JATA                152    0.658 135.371   0.351   0.176   0.004   0.820
E K  1JATA                153    0.616 126.629   0.200   0.053   0.005   0.942
E P  1JATA                154    0.578  82.004  -1.543   0.016   0.005   0.979
E E  1JATA                155    0.941 164.358   0.381   0.000   0.001   0.999

Explain the output.  Go back.
```

---
